# Interferon Gamma +874T/A Polymorphism Increases the Risk of Hepatitis Virus-Related Diseases: Evidence from a Meta-Analysis

**DOI:** 10.1371/journal.pone.0121168

**Published:** 2015-05-04

**Authors:** Yifan Sun, Yu Lu, Taijie Li, Li Xie, Yan Deng, Shan Li, Xue Qin

**Affiliations:** Department of Clinical Laboratory, First Affiliated Hospital of Guangxi Medical University, 6 Shuangyong Road, Nanning, 530021, Guangxi, People's Republic of China; University of Pisa, ITALY

## Abstract

**Background:**

Interferon gamma (IFN-γ) is a key regulatory cytokine, which plays an important role in antiviral defense of an infected host. However, the association between the *IFN*-γ +874T/A gene polymorphism and hepatitis virus-related diseases is heterogeneous.

**Methods:**

Based on the Preferred Reporting Items for Systematic Reviews and Meta-analyses statement, a comprehensive literature search of eligible studies in Embase, Pubmed, and the Cochrane Library was undertaken through November 2014. Odds ratios (ORs) and the corresponding 95% confidence intervals (CIs) were used to measure the strength of the models.

**Results:**

Seventeen case-control articles, including 24 studies with 5503 individuals, met the inclusion criteria. The results indicated a statistically significant association between the *IFN*-γ +874T/A polymorphism and hepatitis virus—related diseases in a recessive gene model (AA vs. TT+TA: OR=1.350, 95% CI=1.101-1.657, *P*=0.004, *I^2^*%=54.3, and *P_Q_*=0.001 for heterogeneity), especially in Asians (OR=1.407, 95% CI=1.035-1.911, *P*=0.029, *I^2^*%=61.9, and *P_Q_*=0.005 for heterogeneity) and hepatitis B virus (HBV)–related disease (OR=1.486, 95% CI=1.195–1.849, *P*=0.000, *I^2^*%=40.4, and *P_Q_*=0.053 for heterogeneity).

**Conclusions:**

The evidence suggests that the *IFN*-γ +874T/A polymorphism increases the risk of hepatitis virus—related diseases, especially in Asians and HBV—related diseases. Further studies on this topic in different ethnicities, especially genome-wide association studies, should be conducted to strengthen our results.

## Introduction

Liver disease can have various causes, including infection with viruses, such as the hepatitis B virus (HBV) and the hepatitis C virus (HCV), especially in developing countries [1]. Interferon gamma (IFN-γ) is a key regulatory cytokine, which plays an important role in antiviral defense by an infected host. IFN-γ can exert antiproliferative and antitumor activity by binding with a specific cell-surface receptor (IFN-γR) [2]. IFN-γ is thought to play a major role in combating chronic hepatitis (CH), liver cirrhosis (LC), and hepatocellular carcinoma (HCC)[3].

**Fig 1:**
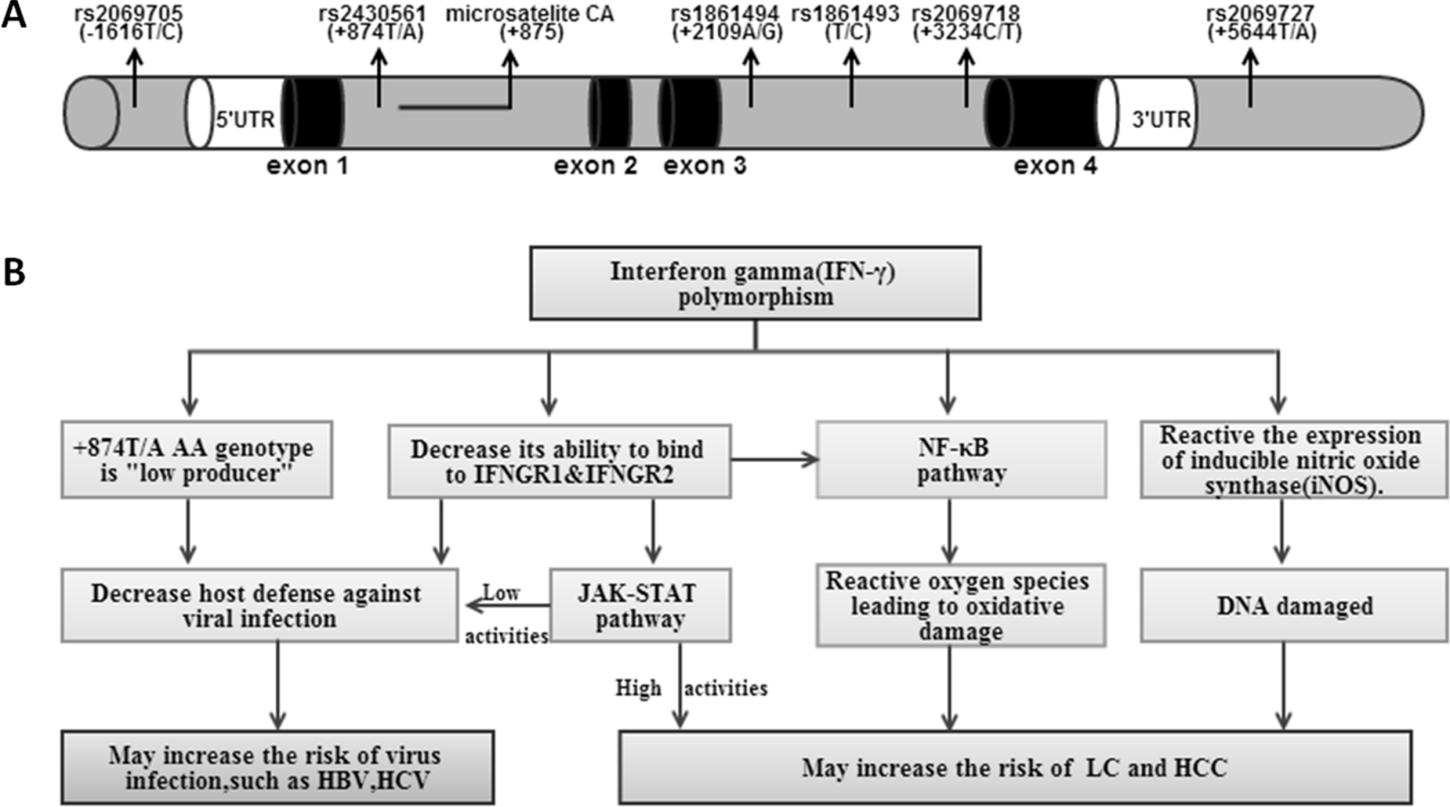
The position of +847A/T (rs2430561) locus and other SNPs in the neighborhood of *IFN-*γ gene on chromosome 12q24(A) and the hypothesis of *IFN-*γ gene polymorphism pathway increase the risk of hepatitis virus-related diseases(B). The figure includes the 5’flanking region (white column), exons (dark column) interrupted by introns (gray column) and 3’flanking region. IFNGR, interferon gamma receptor; JAK, Janus kinase; STAT, Signal Transducer and Activator of Transcription; NF, nuclear factor.

Genetic susceptibility is an important factor in the development of diseases. The *IFN*-*γ* gene on chromosome 12q24 spans approximately 5.4 kb and is composed of four exons, with three intervening regions; many single nucleotide polymorphisms (SNPs) are located in this region [4] (Fig 1). However, only the SNP (+874T/A, rs2430561) of the gene is widely studied. This polymorphism results in the replacement of the T allele with an A allele, which is located at the translation start site in the first intron of the *IFN*-γ gene. The TT genotype is associated with increased IFN production when the immune system responds to stimuli [5]. In contrast, the AA and TA genotypes are linked to low IFN production [5]. The release of IFN-γ by CD4+ Th1 cells, natural killer cells, and other lymphocytes increases T-cell cytotoxicity and natural killer cell activity and enhances cellular immune responses. It appears that the +874T/A polymorphism can influence IFN-γ expression, and in this way, affects immune response.

Many studies have mainly focused on the *IFN*-γ +874T/A polymorphism and virus-related disease risk, especially liver diseases, such as CH, LC, and HCC, which are caused by persistent hepatitis virus infection. However, the literature on the impact of the *IFN*-γ gene polymorphism on the risk of hepatitis virus—related diseases has provided contradictory results [6–16]. Since the *IFN*-γ +874T/A polymorphism is strongly associated with circulating *IFN*-γ concentrations, it is assumed that this polymorphism might be closely related to the risk of the hepatitis virus—related diseases. Our aim was to examine the association between the *IFN*-γ +874T/A polymorphism and the risk of hepatitis virus-related diseases in published studies using a meta-analysis.

## Materials and Methods

This meta-analysis was conducted according to Preferred Reporting Items for Systematic Reviews and Meta-analyses (PRISMA) statement, including the search strategy, selection criteria,data extraction and data analysis [17].

### Identification of eligible studies

We used the following terms to search for articles published in Embase, PubMed, and the Cochrane Library up to November 30, 2014: “interferon,” “IFN” “polymorphism,” “polymorphisms,” “mutation,” “variant,” “hepatitis,” “cirrhosis,” and “hepatocellular carcinoma.” Two investigators (Yifan Sun and Yu Lu) conducted an extensive independent literature search. Articles in reference lists were hand-searched. Only English-language articles and human studies were searched.

### Inclusion and exclusion criteria

The following criteria were used to select suitable studies: (1) case-control or cohort design studies; (2) contained data that could be extracted to calculate the odds ratio (OR), 95% confidence intervals (CIs), and Hardy—Weinberg equilibrium (HWE); (3) stated the DNA genotyping method and source of cases and controls. The following were excluded: review articles, letters, case reports, editorials, conference abstracts, and family-based studies.

### Data extraction

Two investigators (Yifan Sun and Yu Lu) independently extracted data from the included studies. The data included the first author’s name, publication date, country, ethnicity, total sample size, virus type and liver disease type, genotyping method, genotype frequencies of cases and controls, source of the case group and control group, age and sex ratio, source of specimens used to determine the genotypes, and the estimated HWE. If the literature did not provide sufficient data, the investigators tried to contact the author to get the original data by email.

In the subgroup analysis, ethnicities from Europe, West Asia, North Africa, and the Americas were categorized as Caucasian and those from East or Southeast Asia as Asian. The hepatitis virus type and liver disease type were also categorized. If the data in the study were related to various liver diseases, the study was treated as a separate study in the meta-analysis. To determine the accuracy of the extracted information, the data extracted by the two investigators were expected to be the same. In cases of a dispute, they checked their data again. If they could not reach an agreement, the dispute was resolved by a third reviewer (Xue Qin).

### Quality score assessment

Two investigators (Yifan Sun and Shan Li) assessed the quality of the selected studies independently, following the criteria predefined by Thakkinstian et al. [18] and used by Ye et al. [19](S1 Table). The criteria were the sources of the cases and controls, the total sample size, the source of the specimens, and the HWE of the controls. According to the quality score assessment, a study scoring <10 was considered a “low-quality” study, whereas one with a score ≥10 was classified as a “high-quality” study. The lowest score was 0, and the highest score was 15 [19].

### Statistical analysis

The association between the *IFN*-γ +874T/A polymorphism and hepatitis virus-related diseases was assessed with various comparison models, including an allelic model (A vs. T), a co-dominant model (TA vs. TT and AA vs. TT), a dominant model (TA+AA vs. TT), and a recessive model (AA vs. TA+TT). Because the studies lacked details of the environmental factors, unadjusted ORs and the corresponding 95% CIs were used to measure the strength of the models.

In common with previous studies [20, 21], heterogeneity was assessed with a chi-squared Q test and I-squared statistics. If *P*_*Q*_<0.1 or *I* ^*2*^≥50%, the heterogeneity was considered significant. The random-effects model used the DerSimonian and Laird method. Otherwise, the summary OR and the corresponding 95% CI were calculated with a fixed-effects model (the Mantel—Haenszel method). We also conducted a subgroup analysis by ethnicity, genotyping method, source of controls, hepatitis virus type, liver disease type, and quality assessment score. A meta-regression that included the following covariates was performed: ethnicity, genotyping method, source of controls, and quality scores. Covariates with values of *P*<0.05 were considered the main sources of heterogeneity. Galbraith plot analysis was performed for further exploration of the heterogeneity. Sensitivity analysis was conducted to examine such influence by removing studies one by one and recalculating the pooled OR and 95% CI.

A Begg’s funnel plot and an Egger’s test were used to investigate publication bias in the meta-analysis. *P*<0.05 indicated that the result was statistically significant.

All tests in this meta-analysis were conducted with STATA software (version 12.0; Stata Corporation, College Station, Texas, USA).

## Results

### Literature selection and study characteristics

**Fig 2:**
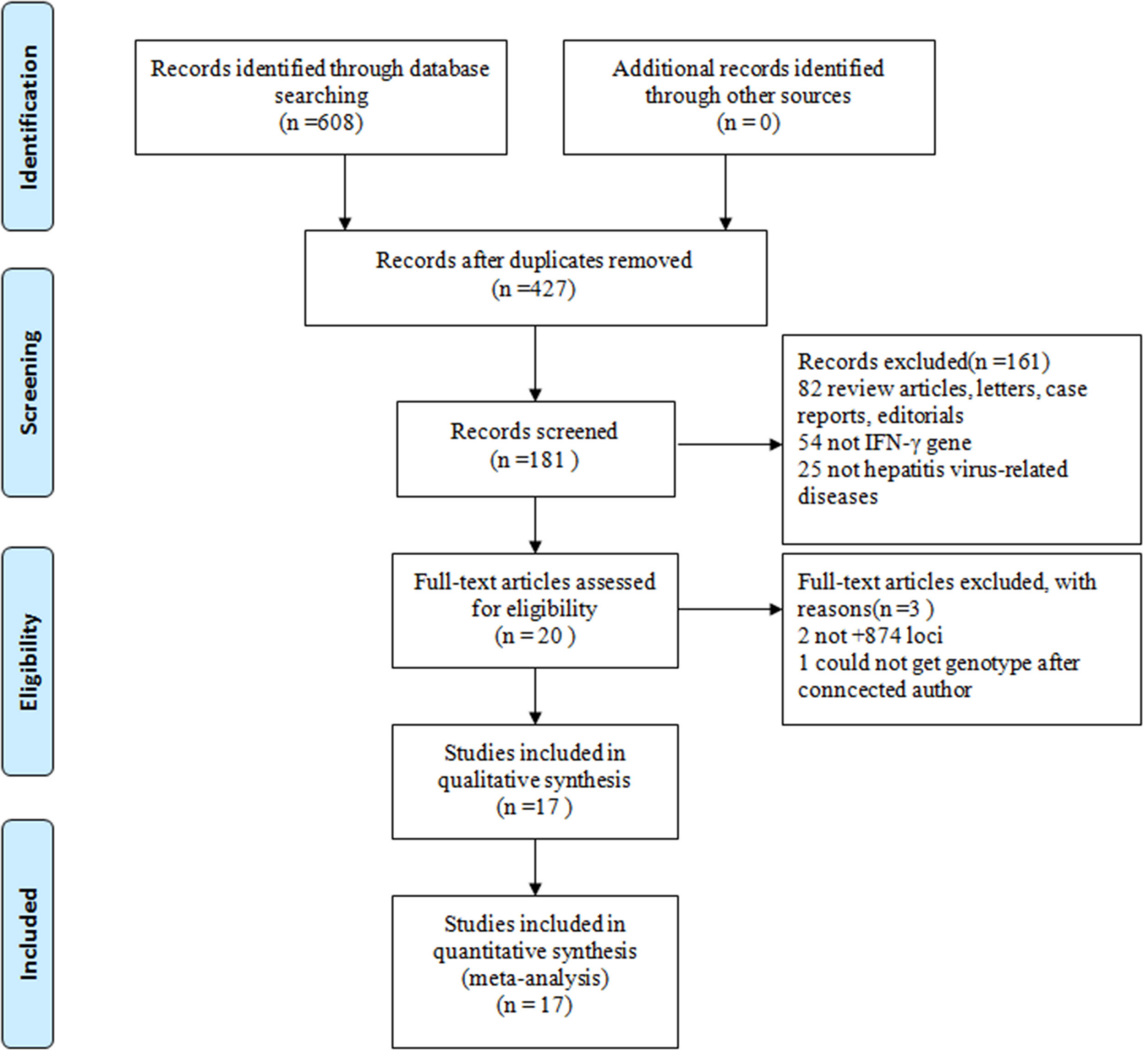
Flow diagram of included studies for this meta-analysis.

Based on the search terms, 17 articles, which included 24 case-control studies containing 2,607 cases and 2,896 controls, were identified as suitable for a meta-analysis [3, 6, 7, 9, 12–14, 16, 22–30] (Fig 2). The primary characteristics of the 24 studies are shown in Table 1. Nine of the articles focused on Caucasians and eight on Asians. Sixteen studies focused on HBV-related diseases, five on HCV-related diseases, one on HDV-related disease, one on HEV-related disease, and one on mixed virus (HBV and HCV)-related HCC. When the articles were categorized by liver disease, two focused on HBV infection (only HBV carrier), nine focused on chronic hepatitis B (CHB), and seven focused on LC/HCC. The genotyping methods included the polymerase chain reaction-amplification refractory mutation system (ARMS-PCR), polymerase chain reaction-sequence specific primers (PCR-SSP), competitively differentiated-polymerase chain reaction (CD-PCR), and DNA sequencing. The HWE of the control population was calculated according to the genotypes. The control population did not fall into HWE in three articles [13, 25, 29]. According to the quality scores, 13 articles were “high-quality” studies. The source of the control population was divided into hospital based and population based.

**Table 1:** Characteristics of Studies Included in This Review.

Study	Year	Country	Enthicity	Liver virus	Liver diseases	Genotyping method	Source of control	Case sequence			Control sequence			*P* * (HWE)	Quality scores
								AA	AT	TT	AA	AT	TT		
Teixeira	2013	Brazil	Caucasian	HBV	HCC	SSP-PCR	PB	40	50	21	79	82	41	0.024	11
Arababadi	2011	Iran	Caucasian	HBV	HBV infection	AMRS-PCR	PB	18	25	14	28	47	25	0.554	9
Basturk	2008	Turkey	Caucasian	HBV	CHB	SSP-PCR	PB	19	23	8	26	24	10	0.282	10
Ben-Ari	2003	Israel	Caucasian	HBV	CHB	SSP-PCR	HB	33	13	10	18	24	6	0.644	10
Ben-Ari	2003	Israel	Caucasian	HBV	LC/HCC	SSP-PCR	HB	17	10	4	18	24	6	0.644	10
Bouzgarrou	2009	Tunisia	Caucasian	HCV	HCV infection	SSP-PCR	PB	22	10	10	33	47	33	0.074	11
Bouzgarrou	2010	Tunisia	Caucasian	HCV	LC/HCC	SSP-PCR	PB	17	21	20	33	47	33	0.074	11
Cheong	2006	South Korea	Asian	HBV	CHB	Sequencing	HB	314	94	5	151	47	3	0.760	11
Conde	2013	Brazil	Caucasian	HBV	CHB	SSP-PCR	PB	27	19	7	51	37	9	0.547	11
Falleti	2007	Italy	Caucasian	HCV	LC/HCC	AMRS-PCR	PB	12	28	10	30	51	15	0.382	11
Gao	2009	China	Asian	HBV	HBV infection	SSP-PCR	PB	25	35	9	14	53	7	<0.000	8
Gao	2009	China	Asian	HCV	HCV infection	SSP-PCR	PB	23	27	5	14	53	7	<0.000	8
Karatayli	2014	Turkey	Caucasian	HDV	CHD	SSP-PCR	HB	9	33	22	11	28	15	0.753	9
Karatayli	2014	Turkey	Caucasian	HBV	CHB	SSP-PCR	HB	37	47	34	11	28	15	0.753	9
Korachi	2013	Turkey	Caucasian	HBV	CHB	Sequencing	PB	38	57	5	16	56	27	0.147	12
Korachi	2013	Turkey	Caucasian	HCV	CHC	Sequencing	PB	20	50	30	16	56	27	0.147	12
Mishra	2011	India	Asian	HEV	HEV infection	Sequencing	PB	24	84	28	90	178	106	0.370	12
Nieters	2004	China	Asian	Mixed	HCC	SSP-PCR	HB	86		155	94		164	>0.200	10
Saxena	2014	India	Asian	HBV	HCC/LC	SSP-PCR	PB	36	56	27	52	77	17	0.180	12
Saxena	2014	India	Asian	HBV	CHB	SSP-PCR	PB	31	20	13	52	77	17	0.180	12
Srivastava	2014	India	Asian	HBV	LC/HCC	SSP-PCR	PB	44	53	29	14	55	7	<0.000	9
Srivastava	2014	India	Asian	HBV	CHB	SSP-PCR	PB	32	61	13	14	55	7	<0.000	9
Peng	2007	China	Asian	HBV	CHB	CD-PCR	HB	247	89	4	65	33	2	0.545	10
Migita	2005	Japan	Asian	HBV	HCC	SSP-PCR	HB	41	7	0	157	31	0	0.218	11

Abbreviation: CHB, chronic hepatitis B;CHD, chronic hepatitis D; LC, liver cirrhosis; HCC, hepatocellular carcinoma; HBV /HCV/HEV infection, only hepatitis virus carriers; SSP-PCR, polymerase chain reaction-sequence specific primers; AMRS-PCR, polymerase chain reaction-amplification refractory mutation system; CD-PCR, competitively differentiated-polymerase chain reaction; HB, Hospital-based; PB, Population-based; Mixed, HBV and HCV infection; HWE, Hardy—Weinberg equilibrium.

*P value of HWE of control

### Allele frequencies in different ethnicities

Allele frequencies in different ethnicities were calculated according to the original data of the studies. The *IFN*-γ +874T/A loci A allele had a higher representation in the controls of the Asian group than in the Caucasian group (65.5% vs. 55.6%, ***χ*** ^**2**^ = 47.07,*P* = 0.000). On average, the frequency of AA, TA, and TT as a proportion of 1 was 0.38, 0.41, and 0.21 respectively in the Asian population controls and 0.32, 0.48, and 0.20 in the Caucasian population; a significant difference was observed between the two ethnicities (***χ*** ^**2**^ = 14.467, *P* = 0.001), as well as in the cases (***χ*** ^**2**^ = 56.53, *P* = 0.000). The allele frequencies in the African group were not analyzed because of the sample size was too small.

### Meta-analysis results

**Fig 3:**
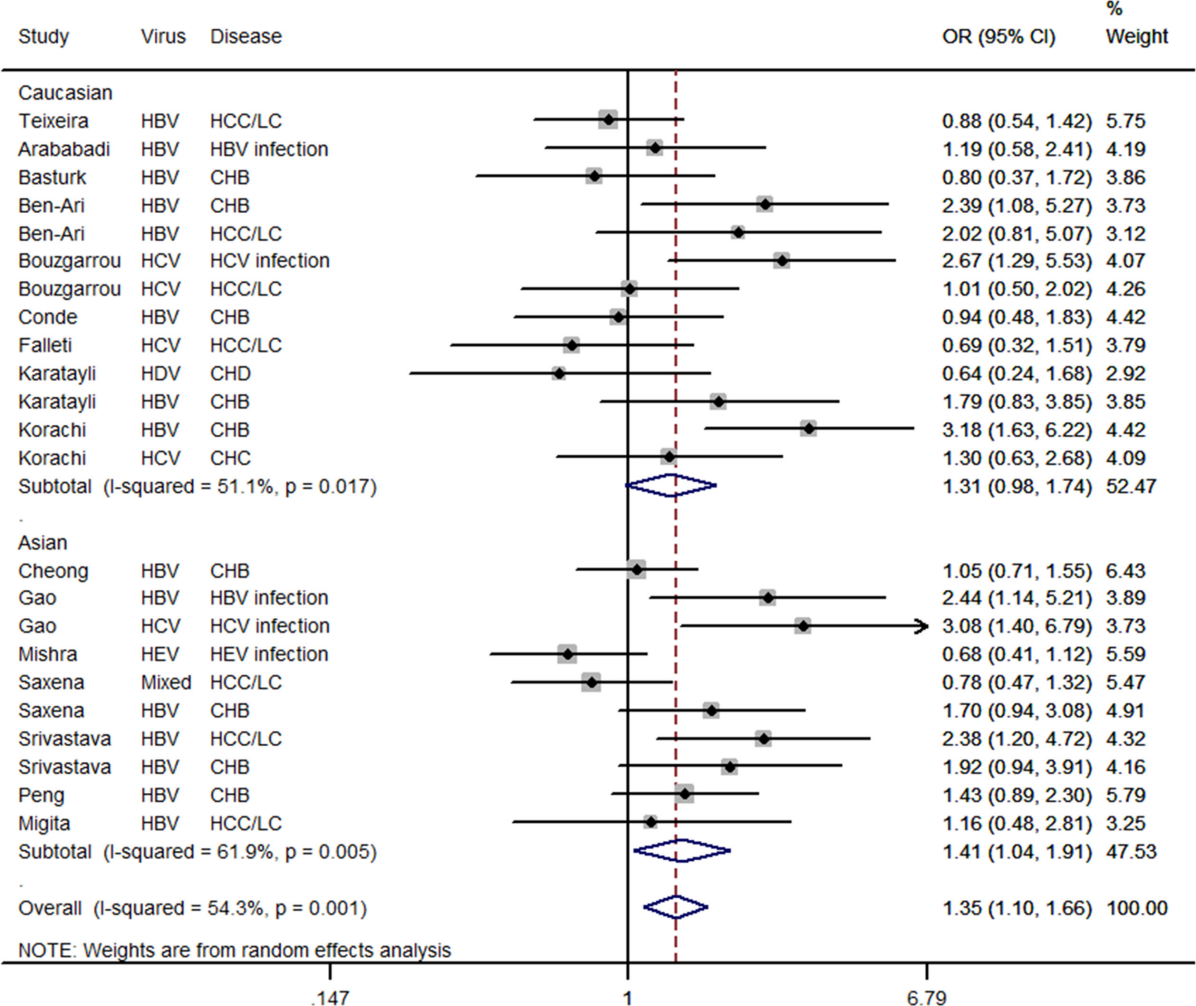
Forest plot for the association between *IFN-*γ +874T/A polymorphism and hepatitis virus-related diseases risk stratified by ethnicity in a recessive model (AA vs. TT+TA) using a random-effects mode. The squares and horizontal lines correspond to the study-specific OR and 95%CI. The diamond represents the summary OR and 95%CI.

The results of the meta-analysis of the *IFN*-γ +874T/A polymorphism and hepatitis virus—related disease risk are listed in Table 2. In the pooled analysis using the random-effects model, there was a significant increased risk of hepatitis virus-related disease in the recessive model (AA vs. TT+TA: OR = 1.350, 95% CI = 1.101–1.657, *P* = 0.004, *I* ^*2*^% = 54.3, and *P*_*Q*_ = 0.001 for heterogeneity; see Fig 3).

**Table 2:** Meta-analysis of interferon gamma +874T/A polymorphisms and hepatitis virus-related diseases.

Group	N	A vs. T		AA vs. TT		TA vs. TT		AA vs. TT+TA		N*	AA+TA vs. TT	
		OR(95%CI)	*P*_*Q*_ ^▲^	OR(95%CI)	*P*_*Q*_ ^▲^	OR(95%CI)	*P*_*Q*_ ^▲^	OR(95%CI)	*P*_*Q*_ ^▲^		OR(95%CI)	*P*_*Q*_ ^▲^
**Overall**	23	1.121(0.993–1.265)	0.022	1.106(0.845–1.446)	0.032	0.789(0.597–1.042)	0.003	**1.284(1.126–1.464)**	0.001	23	0.914(0.738–1.133)	0.031
**Ethnicity**												
Asian	10	1.082(0.953–1.228)	0.246	0.906(0.659–1.246)	0.464	0.634(0.363–1.105)	0.002	**1.407(1.035–1.911)**	0.005	10	0.790(0.559–1.117)	0.051
Caucasian	13	1.134(0.931–1.380)	0.008	1.210(0.820–1.787)	0.013	0.944(0.747–1.194)	0.137	1.306(0.979–1.743)	0.017	13	1.052(0.846–1.308)	0.107
**Virus-related**												
HBV	15	**1.196(1.065–1.343)**	0.169	1.262(0.878–1.815)	0.067	0.763(0.503–1.158)	0.004	**1.486(1.195–1.849)**	0.053	14	0.931(0.651–1.333)	0.020
HCV	5	1.144(0.845–1.550)	0.078	1.182(0.783–1.785)	0.275	0.766(0.525–1.117)	0.999	1.488(0.863–2.565)	0.030	5	0.909(0.643–1.285)	0.869
**Liver disease**												
CHB	9	**1.245(1.009–1.538)**	0.045	1.424(0.791–2.564)	0.012	0.861(0.476–1.557)	0.006	**1.498(1.133–1.980)**	0.070	9	1.063(0.620–1.821)	0.011
LC/HCC	7	0.914(0.774–1.079)	0.547	0.748(0.528–1.059)	0.581	0.623(0.386–1.007)	0.081	1.094(0.782–1.530)	0.068	7	0.751(0.550–1.024)	0.186
**Source of control**												
HB	15	**1.221(1.036–1.438)**	0.652	1.259(0.821–1.933)	0.851	0.692(0.469–1.020)	0.880	**1.475(1.190–1.828)**	0.106	15	0.919(0.709–1.191)	0.988
PB	8	1.070(0.906–1.265)	0.006	1.058(0.738–1.517)	0.004	0.827(0.567–1.205)	0.000	1.235(0.940–1.622)	0.002	8	0.915(0.658–1.273)	0.001
**Score≥10**	16	1.110(0.944–1.304)	0.006	1.111(0.771–1.601)	0.006	0.889(0.621–1.274)	0.003	1.213(0.964–1.526)	0.006	16	0.990(0.753–1.303)	0.014

Abbreviation: N, number of studies; OR, odds ratio; CI, confidence interval; *P*_***Q*,**_ chi-squared Q test value, CHB, chronic hepatitis B;LC, liver cirrhosis;

HCC, hepatocellular carcinoma; HB, Hospital-based; PB, Population-based; Score, quality scores

^▲^If *P*_*Q*_<0.1,using a random-effects model; If *P*_*Q*_≥0.1,using a fixed- effects model.

*number of studies: AA+TA vs. TT mode

**Fig 4:**
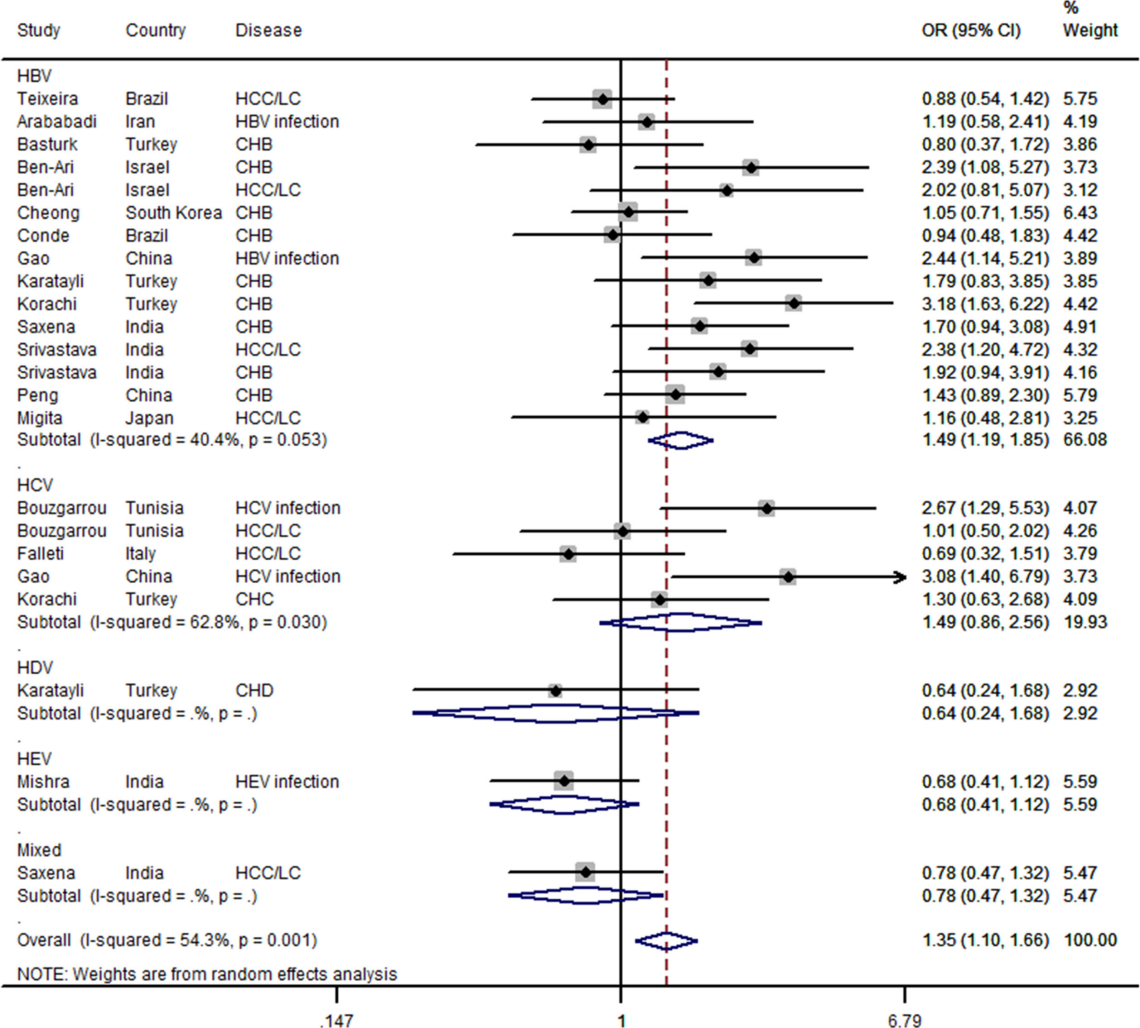
Forest plot for the association between *IFN-*γ +874T/A polymorphism and hepatitis virus-related diseases risk stratified by hepatitis virus type in a recessive model (AA vs. TT+TA) using a random-effects mode. The squares and horizontal lines correspond to the study-specific OR and 95%CI. The diamond represents the summary OR and 95%CI.

For the recessive model, the results of the subgroup analysis showed that the *IFN*-γ +874T/A polymorphism significantly increased the risk of hepatitis virus-related disease in Asians (OR = 1.407, 95% CI = 1.035–1.911, *P* = 0.029, *I* ^*2*^% = 61.9 and *P*_*Q*_ = 0.005 for heterogeneity; see Fig 3). Similar results were found for the HBV—related diseases group (OR = 1.486, 95% CI = 1.195–1.849, *P* = 0.000, *I* ^*2*^% = 40.4, and *P*_*Q*_ = 0.053 for heterogeneity; see Fig 4), CHB patient group (OR = 1.498, 95%CI = 1.133–1.980, *P* = 0.005, *I* ^*2*^% = 44.8, and *P*_*Q*_ = 0.070 for heterogeneity), SSP-PCR method group (OR = 1.449,95%CI = 1.124–1.867, *P* = 0.004, *I* ^*2*^% = 53.8, and *P*_*Q*_ = 0.007 for heterogeneity), and hospital-based population (OR = 1.475, 95% CI = 1.126–1.464, P = 0.000, *I* ^*2*^% = 39.3, and *P*_*Q*_ = 0.106 for heterogeneity).

For the allelic model (allele A vs. allele T), the *IFN*-γ +874T/A polymorphism significantly increased the risk of hepatitis virus—related disease in the HBV-related diseases group (OR = 1.196,95%CI = 1.065–1.343, *P* = 0.003, *I* ^*2*^% = 25.9,and *P*_*Q*_ = 0.169 for heterogeneity). Similar results were observed for the CHB patient group (OR = 1.245, 95% CI = 1.009–1.538, *P* = 0.004, *I* ^*2*^% = 49.0, and *P*_*Q*_ = 0.045 for heterogeneity) and the hospital-based population (OR = 1.221, 95%CI = 1.036–1.438, *P* = 0.017, *I* ^*2*^% = 0.0, and *P*_*Q*_ = 0.652 for heterogeneity).

Other results indicated a lack of statistical significance between the *IFN*-γ +874T/A polymorphism and hepatitis virus—related disease risk (Table 2)

### Heterogeneity analysis

All the models revealed statistical heterogeneity for the *IFN*-γ +874T/A polymorphism in the overall population, with *I* ^*2*^ values of heterogeneity greater than 50% and *P*_*Q*_ values lower than 0.100. Heterogeneity still existed in some studies following the subgroup analysis according to ethnicity, virus genotyping method, sources of control, quality score assessment, hepatitis virus type, and liver disease type. A meta-regression of the sources of heterogeneity revealed that the genotype methods were the main sources of heterogeneity (*P* = 0.005, 95% CI = 0.299–1.470). A Galbraith plot analysis confirmed that the studies by Korachi et al. (HBV), Gao et al. (HCV), Bouzgarrou et al. (HCV), and Mishra et al. (HEV) were responsible for the heterogeneity in the recessive model. After these four studies were excluded, the summary OR value did not change significantly (OR = 1.251, 95% CI = 1.034–1.513, *P* = 0.021, *I* ^*2*^% = 25.8, *P*_*Q*_ = 0.078 for heterogeneity). In the allelic model, the studies by Korachi et al. (HBV) [27] and Saxena et al. (HCC/LC) [3] were the outliers. In the co-dominant model and the dominant model, the summary OR value did not change significantly after these two studies were excluded. However, following their exclusion, the *I* ^*2*^ values were lower than 50%, and the *P*_*Q*_ value was larger than 0.10 (data not shown).

### Sensitivity analysis

The control groups in the studies by Teixeira et al. [29], Gao et al. [25], and Srivastava et al. [14] were out of HWE (Table 1), and these three studies were excluded to perform a sensitivity analysis of the pooled ORs for the *IFN-γ* (+874T/A) polymorphism. Further sensitivity analysis was performed by excluding the studies by Karatayli et al. [26] and Mishra et al. [12], in which the study virus types were HDV and HEV, respectively. Three articles that used the DNA sequencing method to obtain the genotype were also excluded one by one [7, 12, 27]. Finally, the corresponding pooled ORs were not qualitatively altered with or without including these studies (data not shown).

### Publication bias

**Fig 5:**
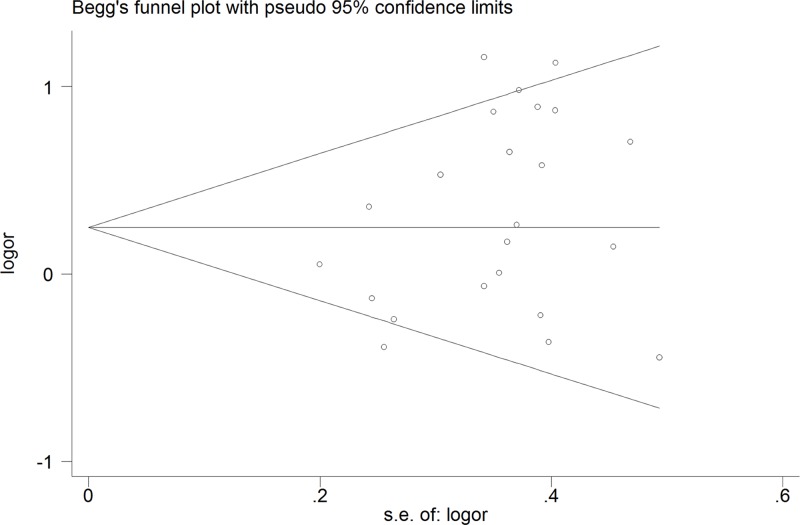
Begg’s funnel plot for contrast in a recessive model (AA vs. TT+TA). Each point represents a separate study for the indicated association. Size graph symbol by weights. *LogOR* natural logarithm of OR. Horizontal line mean effect size.

A Begg’s funnel plot and an Egger’s test were used to investigate the publication bias in the meta-analysis (Fig 5). No significant publication bias was detected with the funnel plot in the overall population in the recessive model. The statistical results of the Egger’s test also provided evidence of funnel plot symmetry (*P*_*Egger’s*_ test = 1.840; *P* = 0.08).

## Discussion

Various environmental and dietary factors are responsible for liver diseases, but hepatitis infection is the main cause of CH, LC, and HCC [31]. The association between the *IFN*-γ +874T/A polymorphism and the risk of hepatitis virus—related diseases has been studied extensively because of the importance of IFN-γ in immune responses [3, 9, 13, 16, 25, 29]. However, previous studies provided inconsistent results. To clarify this issue, we conducted a meta-analysis, and the results showed that there was a significant association between the *IFN*-γ +874T/A polymorphism and risk of hepatitis virus—related disease in the pooled analysis in the recessive model, with the +874 AA genotype associated with a 1.350-fold increased risk of hepatitis virus related—disease, especially in Asian and HBV—related diseases

The results indicate that the *IFN*-γ +874T/A polymorphism may play an important role in hepatitis virus—related disease. The following pathways are considered the main way in which the *IFN*-γ +874T/A polymorphism affects the development of hepatitis virus infection. First, the *IFN*-γ +874T/A genotype TT, which produces a high level of IFN-γ, aids the host’s antiviral defense system. In contrast, the AA and TA genotypes result in low *IFN*-γ production, potentially increasing the risk of hepatitis virus infection [32]. Second, as noted earlier, IFN-γ binds to a specific cell-surface receptor (IFN-γR), which plays a significant role in multiple types of cancers and stimulates cell signaling pathways (JAK-STAT). The *IFN*-γ +874T/A polymorphism leads to dysfunction of IFN-γR, potentially increasing the risk of liver diseases [2]. In addition, the DNA sequence containing the +874 T allele is the specific binding site for the nuclear factor Kappa B(NF-*κ*B) transcription factor. If the NF-κB pathway is affected, it may lead to oxidative damage, which can also increase the risk of LC and HCC [32]. The possible pathway of the *IFN*-*γ* polymorphism increase in hepatitis virus—related diseases risk is shown in Fig 1.

The stratified analysis by ethnicity in the present study suggested that the *IFN*-γ +874T/A polymorphism influenced the risk of hepatitis virus-related diseases in Asians but not in Caucasians. Many factors may contribute to this finding. First, hepatitis virus related—disease is a complex entity, with multiple determinants other than virus infection, such as diet and the environment. Some studies noted significant gene—gene and gene—environmental interactions in patients with hepatitis virus related—diseases [33–35]. Second, as shown in previous studies, the gene frequency of the *IFN-*γ +874T/A polymorphism varies in ethnicities [36, 37], and the Asian population had a higher rate of the A allele than that in the Caucasian population in our study. Third, the inclusion of study subjects from different populations, together with the various genotyping methods and the different sample sizes used in these studies, means it is not possible to reach a definitive conclusion, and the results remain conflicting.

The subgroup analysis showed that the significant results are mainly reflected in the HBV—related group. Evidence suggests that IFN-γ can suppress viral replication/clear HBV without causing liver injury, the non-cytolytic HBV suppression effect of IFN-γ is believed to have an important role in the control of HBV viral activity in patients with CHB [38]. Regarding CHB, *IFN-γ* +874 was seen to play a functional role in relation to viral load, consistent with its known role inhibiting and replicating of HBV-infected cells [16]. IFN-γ can also induce expression of HLA class II [39], and a genome-wide association study (GWAS) showed that HLA contributes to the risk of persistent HBV infection [40].

As we now known, the *IFN*-γ +874T/A TT genotype produces a high level of IFN-γ, and the AA and TA genotypes result in low IFN-γ production. Thus we can suppose that the AA+TA vs. TT model may have significant association in the analysis. However, the ORs of AA vs. TT+TA and AA+TA vs. TT are opposite in our meta-analysis. These confusing results suggested that the T allele is a protective gene in liver disease; moreover, this allele has more power than the A allele, therefore, only the population with the AA genotype has higher risk of hepatitis virus-related liver disease. Indeed, similar results happened in the association between the *IFN*-γ +874T/A polymorphism and cervical cancer with human papillomavirus (HPV) infection [41] and leprosy caused by *Mycobacterium leprae*[42]. A meta-analysis also indicated that the +874 T allele of *IFN*-γ showed a protective significant association with tuberculosis susceptibility [43].

Studies have also reported the role of the *IFN*-γ gene in the response to drug treatment in cancer [44]. Sarvari et al. [13] indicated that the +2109 locus of the *IFN*-γ gene, which is also the specific binding site for NF-*κ*B similar to the +874 locus, influenced HCV therapy. Moreover, Oxenkrug et al. [45] found that T alleles at the +874 locus of the *IFN*-γ gene were a risk factor in depressed patients with HCV who were treated with IFN-alpha. The *IFN-*γ gene polymorphism may further affect the development of HCC and the response to cancer drugs by increasing the risk of hepatitis virus-related diseases, although we did not observe a significant association between the (+874T/A) polymorphism and HCC risk in this study, which is worthy of further investigation in the future.

We noticed that the meta-analysis by Ge et al.[46] also suggested that the +874T/A polymorphism may not contribute to HCC susceptibility with three studies, which was verified by our meta-analysis with six studies. In addition, Sun et al. [47] also performed a meta-analysis of the +874T/A polymorphism and CHB risk with five studies, and their results indicated the TT genotype and the T allele reduce the risk of chronic HBV infection in Asian individuals, which was similar to our meta-analysis of nine studies. However, we did not observed a significant association in co-dominant models. These differences may be because more studies were involved in our study, therefore; therefore, we believe our results are more robust with more statistical power.

Heterogeneity is the most common problem when explaining the results of a meta-analysis. In the current meta-analysis, we assessed heterogeneity using various statistical methods, including subgroup analysis, meta-regression, and Galbraith plot analysis. Finally, we concluded that the heterogeneity in the meta-analysis was due to the genotyping method, mixed infection, and various virus types. Sensitivity analysis was performed by excluding the studies not in HWE. The studies of HDV infection and HEV infection and those that used different genotyping methods, especially the DNA sequencing method, were also excluded one by one. The corresponding pooled OR value did not differ significantly from that of the overall meta-analysis. However, a Begg’s funnel plot and an Egger’s test (*P*>0.05) showed no publication bias. Therefore, we consider that the results are credible, although there are some confounding factors in the articles.

GWASs have evolved over the last 10 years into a powerful tool for investigating the genetic architecture of human disease. Recently, many GWASs determined that SNPs at *HLA-DPA1*, *HLA-DPB1*, *HLA-DQB1–HLA-DQA2* and *HLA-DQB2* are associated with chronic HBV infection in Japanese and Korean populations [40, 48, 49], and Hu et al. identified two new loci associated with chronic HBV infection: rs3130542 at 6p21.33 and rs4821116 at 22q11.21 in Han Chinese [50]. In addition, rs17401966 in *KIF1B* on chromosome 1p36.22 locus confers susceptibility to HBV-related HCC [51]. Our results suggested that rs2430561 in *IFN*-γ on chromosome 12q24 contributes to susceptibility to HBV-related diseases based on traditional genotyping methods, and these results have motivated us to carry out a GWAS focus on the SNPs on chromosome 12 to discover novel susceptibility loci for HBV-related diseases in the future.

Several limitations of the present meta-analysis should be considered when interpreting the results. First, most of the included studies had small sample sizes, which led to insufficient statistical power to explore the interaction between the *IFN-*γ +874T/A polymorphism and hepatitis virus-related diseases. The lack of association in the other ethnicity or genetic model may be most likely because of insufficient studies. Further studies on this topic in different ethnicities are expected to be conducted to strengthen our results. Second, because a lack in the original data of the reviewed studies, a more precise analysis could not conducted of individual information including other covariates such as age and sex. In spite of these, our meta-analysis had some advantages. First, a substantial number of cases and controls were pooled from different studies, which greatly increased the statistical power compared with individual studies. Second, the results of the sensitivity analyses were not materially altered and did not draw different conclusions, indicating that our results were moderately robust.

## Conclusion

In summary, this meta-analysis indicated that the *IFN-*γ +874T/A polymorphism increases the risk of hepatitis virus—related diseases, especially in Asians and HBV-related disease. We expect more relevant studies to be published on this topic in the future to strengthen our conclusion, as it remains a topic of concern and interest.

## Supporting Information

S1 Table Scale for quality assessment. (DOC)

S2 Table PRISMA 2009 Checklist. (DOC)

S3 Table Meta-analysis on Genetic Association Studies Checklist| PLOS ONE. (DOCX)
