# Supplementary material for: Interferon Gamma +874T/A Polymorphism Increases the Risk of Hepatitis Virus-Related Diseases: Evidence from a Meta-Analysis
Source: PLoS One. 2015 May 4;10(5):e0121168. doi: 10.1371/journal.pone.0121168 (PMC4418602; doi:10.1371/journal.pone.0121168)
Supplement: S1 Table — (DOC) [file pone.0121168.s001.doc]

| **Table 1** **Scale for quality assessment** | |
| --- | --- |
| **Criteria** | **Score** |
| Reprtiesentatieness of cases |  |
| Selected from population or cancer registry | 3 |
| Selected from hospital | 2 |
| Selecte from pathology archives bu without description | 1 |
| Not described | 0 |
| Reprtiesentatieness of controls |  |
| Population-based | 3 |
| Blood donors or volunteers | 2 |
| Hospital-based(cancer-free patients) | 1 |
| Not described | 0 |
| Specimens of cases determining genotypes |  |
| White blood cells or normal tissues | 3 |
| Tumor tissues or exfoliated cells of tissue | 0 |
| Hardy-weinberg equilibrium in controls |  |
| Hardy-weinberg equilibrium | 3 |
| Hardy-weinberg disequilibrium | 0 |
| Total sample size |  |
| ≥1000 | 3 |
| ≥500 but <1000 | 2 |
| ≥200 but <500 | 1 |
| <200 | 0 |
